# Characteristics and Work Life Quality of Nursing Home Care Aides in Canada

**DOI:** 10.1001/jamanetworkopen.2020.29121

**Published:** 2020-12-09

**Authors:** Yuting Song, Ala Iaconi, Stephanie A. Chamberlain, Greta Cummings, Matthias Hoben, Peter Norton, Carole Estabrooks

**Affiliations:** 1 Faculty of Nursing, University of Alberta, Edmonton, Alberta, Canada; 2 Department of Family Medicine, Faculty of Medicine and Dentistry, University of Alberta, Edmonton, Alberta, Canada; 3 Department of Family Medicine, Faculty of Medicine, University of Calgary, Calgary, Alberta, Canada

## Abstract

This cross-sectional study describes care aides’ characteristics and quality of work life in Western Canadian nursing homes.

## Introduction

The need for profound, systemic change in the nursing home sector has been clear for decades.^1^ The coronavirus disease 2019 pandemic has exacerbated existing deficiencies in the sector.^2,3^ Care aides (called certified nursing assistants in the US) provide up to 90% of direct care in Canadian nursing homes.^4^ They are both a neglected and socioeconomically disadvantaged workforce, as well as a critical source of emotional and social support for residents.^4,5^ Our objective is to describe care aides’ characteristics and quality of work life in Western Canadian nursing homes.

## Methods

This is a cross-sectional analysis of care aide survey data collected between September 3, 2019, and February 28, 2020.^4^ The study was approved by research ethics boards at University of Alberta, University of British Columbia, and University of Manitoba. Operational approvals were obtained from participating organizations. Participants completed written informed consent. This study follows the Strengthening the Reporting of Observational Studies in Epidemiology (STROBE) reporting guideline.

Care aides were from urban facilities, selected according to stratified random sampling (health region, owner-operator, and bed size). Trained interviewers collected data using structured interviews.^4^ Eligible care aides had worked in a facility more than 3 months, could identify a unit where they worked for at least 50% of their time, and had worked on that unit for 6 or more shifts in the past month.

For categorical variables, we calculated the frequencies and percentages. For normally distributed continuous variables, we calculated means and SDs; otherwise, medians and interquartile ranges were calculated. Data analysis was performed from April to May 2020.

## Results

Of 5381 eligible care aides in 91 nursing homes, 3765 (69.97%) participated in the study. Care aides were predominantly women (3359 aides [89.22%]), aged 40 years or older (2632 aides [69.91%]), and spoke English as an additional language (2571 aides [68.29%]). Approximately 25% (915 aides [24.30%]) reported working in more than 1 nursing home (Table 1).

**Table 1.** Characteristics of Nursing Home Care Aides

Characteristic	Care aides, No. (%) (N = 3765)
Age, y	
<30	318 (8.45)
30-39	815 (21.65)
40-49	1186 (31.50)
50-59	1019 (27.07)
≥60	427 (11.34)
Female	3359 (89.22)
Care aide certificate	3526 (93.65)
English as an additional language	2571 (68.29)
Shift worked most of the time	
Day	1909 (50.70)
Evening	1381 (36.68)
Night	475 (12.62)
Experience, median (interquartile range), y	
On unit	4 (1.33-9.50)
As care aide	10 (5-17)
Nursing homes worked in, No.	
1	2848 (75.64)
≥2	915 (24.30)

More than 70% of care aides reported moderate to high risk for emotional exhaustion (2718 aides [72.19%]) and cynicism (3146 aides [83.56%]), which are 2 core indicators of burnout (Table 2). Approximately one-half of aides (1890 aides [50.20%]) reported that they had to work short-staffed daily or weekly in the past month. They reported frequently rushing and missing essential care tasks in their most recent shift and often experienced responsive behaviors from residents associated with dementia. Less than 30% (960 aides [25.51%]) reported being frequently engaged in team meetings about residents, and less than 5% (179 aides [4.76%]) were frequently engaged in family conferences. Nevertheless, care aides reported feeling satisfied with their jobs. The majority had high levels of professional efficacy and psychological empowerment.

**Table 2.** Work-Related Outcomes for Nursing Home Care Aides

Variables	Care aides, No. (%)
Responsive behaviors experienced from residents in last 5 shifts	
Yelling and screaming	3240 (86.06)
Hurtful remarks or behaviors	2742 (72.83)
Being spit on, bitten, hit, pushed, or pinched	2392 (63.53)
Verbal threats	2351 (62.44)
Repeated and unwanted questions or remarks of a sexual nature	918 (24.38)
Sexual touching	481 (12.78)
Risk of burnout symptoms^a^	
Emotional exhaustion	
High	1607 (42.68)
Moderate	1111 (29.51)
Low	1047 (27.81)
Cynicism	
High	1906 (50.62)
Moderate	1240 (32.93)
Low	619 (16.44)
Professional efficacy	
High	95 (2.52)
Moderate	302 (8.02)
Low	3368 (89.46)
Feeling work short-staffed in the last month	
Daily	604 (16.04)
Weekly	1286 (34.16)
Monthly	576 (15.3)
Less often	842 (22.36)
Never	453 (12.03)
Care tasks rushed on most recent shift	
Talking with residents	1912 (50.78)
Toileting	1716 (45.58)
Mouth care	1597 (42.42)
Feeding residents	1566 (41.59)
Bathing residents	1395 (37.05)
Care tasks undone due to lack of time on most recent shift	
Taking residents for a walk	1538 (40.85)
Talking with residents	1293 (34.34)
Mouth care	586 (15.56)
Toileting	370 (9.83)
Bathing	348 (9.24)
Feeding	241 (6.40)
Engagement in decision-making meetings	
Team meetings about residents	960 (25.51)
Family conferences	179 (4.76)
Change of shift report	3121 (82.94)
Job satisfaction, mean (SD)^b^	4.25 (0.64)
Psychological empowerment, mean (SD)^c^	
Competence	4.51 (0.47)
Meaning	4.56 (0.49)
Self-determination	4.06 (0.72)
Impact	3.75 (0.69)

^a^ A high risk for burnout is indicated by 1 or more of the following: emotional exhaustion score of greater than 3.00, cynicism score greater than 2.33, and efficacy score less than 3.30. A low risk for burnout is indicated by 1 or more of the following: emotional exhaustion score less than 1.67, cynicism score less than 1.00, and efficacy score greater than 4.00. The score range for emotional exhaustion, cynicism, and efficacy is 0 (never) to 6 (daily), with higher scores indicating higher levels of all 3.

^b^ The score range for job satisfaction is 1 (strongly disagree) to 5 (strongly agree), with a higher score indicating a higher level of job satisfaction.

^c^ Psychological empowerment reflects an active orientation in which an individual wishes and feels able to shape their work role and context. The score range is 1 (strongly disagree) to 5 (strongly agree) with a higher score indicating a higher level of psychological empowerment.

## Discussion

Most care aides are middle-aged to older women who speak English as an additional language. Although they are highly satisfied with their jobs, they work in a resource-constrained environment (eg, often had to rush or miss care tasks). These findings, compared with our previous report,^4^ suggest that these prepandemic conditions were stable over a relatively long period (2009-2020). However, despite stability, our findings indicate a workforce under strain—an at-risk group caring for an even more at-risk resident group^5^—a perfect storm for crisis, as the world has observed.^2,3^

Care aides are uniquely positioned to make significant contributions to improving resident care quality and quality of life. Our ongoing work demonstrates that care aide–led improvement programs achieve sustained positive impact on residents’ clinical outcomes, including pain, mobility, and responsive behaviors.^6^ As one care aide said, “The nurses listen to all the ideas instead of going straight to medication when a simple touch or letting them sleep would have solved the problem. There is more interaction. Some little things can help instead of having to resort to medications.”^6^ Generalizations of these findings to care aides with characteristics different from those in urban areas of Western Canada should be made with caution. Survey responses are subject to the usual cautions when interpreting findings (e.g., self-report biases).
